# Organic Derivatives of Mercury and Tin as Promoters of Membrane Lipid Peroxidation

**DOI:** 10.1155/S1565363304000068

**Published:** 2004

**Authors:** E. Milaeva, V. Petrosyan, N. Berberova, Y. Pimenov, L. Pellerito

**Affiliations:** 1 Department of Organic Chemistry Moscow State Lomonosov University Lelinsky Gory Moscow 119899 Russia; 2 Department of Organic and Biological Chemistry Astrakhan' State Technical University Tatischev str. 16 Astrakhan' 414025 Russia; 3 Department of lnorganic and Analytical Chemistry University of Palermo viale delle Scienze 2 Parco d'Orleans Palermo I-90128 Italy

## Abstract

The toxicity mechanisms of mercury and tin organic derivatives are still under debate. Generally the presence of organic moieties in their molecules makes these compounds lipophilic and membrane active species. The recent results suggest that Hg and Sn compounds deplete HS-groups in proteins, glutathione and glutathione-dependent enzymatic systems; this process also results in the production of reactive oxygen species (ROS), the enhancement of membrane lipids peroxidation and damage of the antioxidative defence system. The goal of this review is to present recent results in the studies oriented towards the role of organomercury and organotin compounds in the xenobiotic-mediated enhancement of radical production and hence in the promotion of cell damage as a result of enhanced lipids peroxidation. Moreover the conception of the carbon to metal bond cleavage that leads to the generation of reactive organic radicals is discussed as one of the mechanisms of mercury and tin organic derivatives toxicity. The possible use of natural and synthetic antioxidants as detoxification agents is described. The data collected recently and presented here are fundamentally important to recognizing the difference between the role of metal center and of organic fragments in the biochemical behavior of organomercury and organotin compounds in their interaction with primary biological targets when entering a living organism.


### Organic Derivatives of Mercury and Tin as Promoters of Membrane Lipid Peroxidation

E.Milaeva (✉) · V.Petrosyan
*Department of Organic Chemistry, Moscow State Lomonosov University, Lelinsky Gory, Moscow, 119899 Russia*
E-mail: milaeva@org.chem.msu.su; Tel.: +7 (095) 9393864
N. Berberova · Y. Pimenov
*Department of Organic and Biological Chemistry, Astrakhan' State Technical University, Tatischev str. 16, Astrakhan', 414025 Russia*
L. Pellerito
*Department of Inorganic and Analytical Chemistry, University of Palermo, viale delle Scienze 2, Parco d'Orleans, Palermo, I-90128 ITALY*

**ABSTRACT**

The toxicity mechanisms of mercury and tin organic derivatives are still under debate. Generally the presence of organic moieties in their molecules makes these compounds lipophilic and membrane active species. The recent results suggest that Hg and Sn compounds deplete HS-groups in proteins, glutathione and glutathione-dependent enzymatic systems; this process also results in the production of reactive oxygen species (ROS), the enhancement of membrane lipids peroxidation and damage of the antioxidative defence system. The goal of this review is to present recent results in the studies oriented towards the role of organomercury and organotin compounds in the xenobiotic-mediated enhancement of radical production and hence in the promotion of cell damage as a result of enhanced lipids peroxidation. Moreover the conception of the carbon to metal bond cleavage that leads to the generation of reactive organic radicals is discussed as one of the mechanisms of mercury and tin organic derivatives toxicity. The possible use of natural and synthetic antioxidants as detoxification agents is described. The data collected recently and presented here are fundamentally important to recognizing the difference between the role of metal center and of organic fragments in the biochemical behavior of organomercury and organotin compounds in their interaction with primary biological targets when entering a living organism.

**Keywords:** Organometallic compounds · Mercury · Tin · Membrane · Lipids peroxidation · Radicals · Oleic acid · Antioxidants

**Abbreviations:** GST: glutathione S-transferase · DMPC: dimyristoyl-L-α-phosphatidylcholine · MDA: malonic dialdehyde · PC: phosphocholine · ROS: reactive oxygen species · TBARS: thiobarbituric acid reactive substances

### INTRODUCTION

Due to the widespread use of the organometallic compounds $R_nMX_m$, in particular organomercurials RHgX, $R_2Hg$ and organotins $R_nSnX_{4-n}$ (n = 0 - 4), a considerable amount of these highly toxic xenobiotics enters various ecosystems /1/. The accumulation of $R_nMX_m$ in biota leads to a phenomenon relevant to toxicants transfer to higher organisms and therefore their extremely hazardous impact on human health and on the environment is a matter of great concern /2,3/.

Within the class of organometallic ecotoxicants, $R_nMX_m$, there are considerable variations in toxicity depending both on the nature of metal atom M, number and nature of the organic groups R and the nature of species formed in various media. Generally the presence of organic moieties in their molecules makes these compounds lipophilic and membrane active species /4,5/. Organic derivatives of Hg and Sn are supposed to induce membrane associated oxidative stress in living organism through different mechanisms including the possibility to enhance the intracellular generation of reactive oxygen species, $H_2O_2$, $O_2^{-\bullet}$, $HO^\bullet$ (ROS) /6-8/.

It is well known that Hg and Sn compounds deplete HS-groups in proteins and glutathione; this process also results in the production of ROS /7,9/. Enhanced lipid peroxidation, DNA and sulfhydryl homeostasis damage, the decrease of total glutathione level, the inhibition of superoxide dismutase, catalase, glutathione reductase, Se-dependent and Se-independent glutathione peroxidases, glutathione S-transferases and perturbation of antioxidant defense system are the consequences of this impact /10,11/. However, little is known about the molecular mechanisms of organometallics action as exogeneous prooxidant stressors. To understand the biomolecular mode of organomercury and organtin compounds action as promoters of cellular oxidative stress, the participation of various $R_nMX_m$ in key biochemical processes responsible for the extensive lipids peroxidation and damage of the antioxidative defence system should be studied.

The interaction of organic derivatives of heavy metals with free sulfhydryl groups in proteins that leads to the metal-induced cell death is well known /12/. The involvement of $R_nMX_m$ (with general formula $R_nM$) in oxidative/free radical reactions, namely in radical chain oxidation of the biological substrate R'H, is purely investigated (Fig. 1). The interaction of $R_nM$ may include the reactions either with peroxyl radicals $R'OO^\bullet$ or with hydroperoxides R'OOH that are main intermediates of R'H oxidation and lead to the homolytic cleavage of C-M bonds and result in the formation of reactive organic radicals $R^\bullet$ responsible for the enhanced lipid peroxidation and cell death.

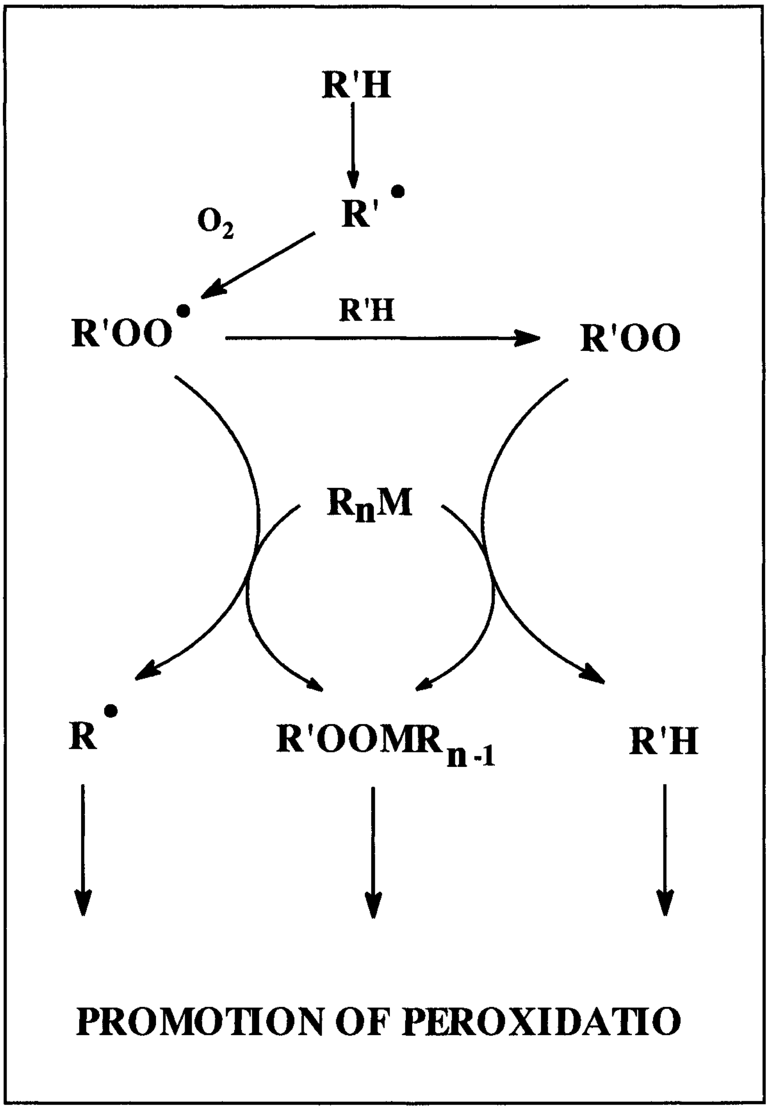

**Fig. 1**: The involvement of organometallic compounds $R_nM$ [$R_nM$ – general formula for RHgX, $R_2Hg$, $R_nSnX_{4-n}$ (n = 0 - 4)] in the interaction with peroxyl radicals R'OO• and hydroperoxides R'OOH as key intermediates of substrate R'H oxidation.

The goal of this review is to present recent results in the studies oriented towards the role of organomercury and organotin compounds in the xenobiotic-mediated enhancement of radical production and hence in the promotion of cell damage as a result of enhanced lipids peroxidation. Moreover the conception of the carbon to metal bond cleavage will be discussed as one of the mechanisms of mercury and tin organic derivatives cytotoxcity and the possible use of natural and synthetic antioxidants as detoxification agents will be described.

### TOTAL LIPIDS CONTENT AS A BIOMARKER OF ORGANOMETALLICS INDUCED OXIDATIVE MEMBRANE DAMAGE

Xenobiotic-induced lipids peroxidation, as a critical stage in toxicological processes, describes the non-enzymatic oxidative destruction of fats /11/. The peroxidation of unsaturated fatty acids (oleic, linoleic, linolenic, arachidonic acids) in membrane lipid bilayer leads to the membrane cells damage /9/. The decrease of the membrane fluidity and potential, permeability to $H^+$ and $Ca^{2+}$ ions is observed as a result of lipids peroxidation.

It has been observed that the presence of 0.15 ppb $[(n\text{-}C_4H_9)_3Sn]_2O$ caused a highly significant decrease in the total lipids content in body tissues (digestive gland, gills and foot) of the estuarine edible clam, *Anadara rhombea* /13/. Acute (0.05 ppb) and chronic (0.02 ppb) exposures to $[(n\text{-}C_4H_9)_3Sn]_2O$ induced various changes in vital biochemical systems in *A. rhombea*. The change of the lipids content might be explained as a consequence of the degradation of the unsaturated fatty acids mediated by organotins. The influence of $(C_6H_5)_3SnCl$ and $(C_6H_5)_2SnCl_2$ on the fatty acids composition in a marine chlorophyte, *Dunaliella tertiolecta* Butcher and a marine diatom, *Skeletonema costatum (Greville) Cleve*, which exhibit different resistances to phenyltins, were studied in the period of 72 h /14/. The proposition that sensitivity to phenyltin compounds is related to their fatty acid composition has been confirmed when attempts were made to grow *D. tertiolecta* in the presence of $(C_6H_5)_3SnCl$ ranging in concentration from $2.1\cdot10^{-3}$ to $2.1\cdot10^{-1}$ μM, and *S. costatum* in the presence of $(C_6H_5)_3SnCl$ and $(C_6H_5)_2SnCl_2$ in concentrations ranging from $8.4\cdot10^{-5}$ to $8.4\cdot10^{-3}$ μM and $8.4\cdot10^{-3}$ to $8.4\cdot10^{-1}$ μM respectively. The results show a 45% increase in monounsaturated fatty acids with a decrease in total polyunsaturated fatty acids that are more easily oxidized substrates.

Enhanced lipid peroxidation in liver, kidney and brain of mice has been observed after exposure to $CH_3HgCl$ (10-40 mg/l in drinking water) for 2 weeks /15/. An increase in catalase and glutathione S-transferase (GST) activities was observed in the sheaths of the marine phanerogam *Posidonia oceanica (L.) Delile* experimentally exposed to inorganic mercury /16/ indicating that the antioxidant mechanisms were overtaxed and could not prevent membrane lipid peroxidation. The effect of $HgCl_2$ on lipid profiles in organs of freshwater cat-fish (*Heteropneustes fossilis*) was studied /17/. The daily exposure of $HgCl_2$ 0.2 mg/L for 10, 20 and 30 days depleted the total lipids in brain. The content of phospholipids enhanced significantly in 30 days. Liver exhibited elevated levels of total lipids. Kidney showed a marked decrease in the content of total lipids at higher exposure to $HgCl_2$. The content of total lipids and phospholipids was high in muscle.

The membrane lipids composition markedly influences membrane permeabilisation /18/. The direct toxic effect of $(n\text{-}C_4H_9)_3SnCl$ on growth of ***Saccharomyces cerevisiae*** was inhibited by the enrichment of cells with linoleic acid. Since the cellular response to organometallic compounds exposure might be influenced by the supplementation with fatty acids the stability of organs and tissues is expected to depend on their total content in lipids /19/.

The effect of $(n\text{-}C_4H_9)_3SnCl$ observed in the study of two species of amphipods, *Rhepoxynius abronius Barnard* (*Phoxacephalidae*) and *Eohaustorius estuarius Bosworth* (*Haustoriidae*), commonly used in sediment bioassays, showed that the decrease in whole-body lipid content may be an indicator of declining animal health (and increased sensitivity to toxicants) /20/.

A comparative study on the interactions between $R_2SnCl_2$, $R_3SnCl$ (R = alkyl or phenyl groups) and model bilayer lipid membranes has been performed using the relative degree of depolarization of the lipid membrane potential as a parameter of the toxicity /21/. The high lipophilic $R_3SnCl$ were the most active species. It was shown that a correlation exists between depolarization activity and the lipophilicity of $R_3SnCl$ hydrolysis products. The surface charge of modified membranes had a secondary influence on depolarization efficiency of organotin compounds /22/. The interaction of $(n\text{-}C_4H_9)_3SnCl$ and $(C_6H_5)_3SnCl$ with model membranes composed of different phosphatidylethanolamines has been studied by means of differential scanning calorimetry, X-ray diffraction, NMR $^{31}P$ and IR spectroscopy /23/. It has been shown that $(n\text{-}C_4H_9)_3SnCl$ and $(C_6H_5)_3SnCl$ segregate in phosphatidylethanolamine membranes and disrupt the pattern of H-bonding in the interfacial region of dielaidoylphosphatidylethanolamine membranes. The authors proposed that the specific toxic effects of organotins might be exerted through the alteration of membrane function produced by interaction of $(n\text{-}C_4H_9)_3SnCl$ and $(C_6H_5)_3SnCl$ with the lipids component of the membrane.

The results of various experimental studies show that the degradation of lipids is one of the possible mechanisms involved in organometallics toxicity.

### PEROXIDATION OF UNSATURATED FATTY ACIDS IN THE PRESENCE OF ORGANOMETALLICS

The influence of organometallic compounds RHgX, $R_2Hg$, $R_nSnX_{4-n}$ bearing various organic groups R upon the lipid peroxidation level was studied using unsaturated fatty acids model compounds – oleic acid and methyl oleate as substrates R'H /24-27/.

The monitoring was performed at various temperatures by measuring the total concentration of isomeric hydroperoxides R'OOH and the concentration of thiobarbituric acid reactive substances (TBARS), namely malonic dialdehyde (MDA), as a marker of the carbonyl compounds formation following the hydroperoxides decomposition /28/. The difference in substrate modification (oleic acid or its methyl ester) does not affect the kinetic data of R'OOH formation /24/; therefore the interaction of organometallic compounds with the carboxylic group in oleic acid might be omitted.

The kinetic data for the oleic acid oxidation (rate constants of R'OOH accumulation k and relative change in R'OOH concentrations $A_i$) in the presence of organomercury and organotin compounds presented in Table 1 show the dependence of R'OOH formation both on the nature of metal (M) in $R_nMX_m$ and temperature. The increase of R'OOH concentration has been observed in the presence of all organotins under investigation at 25, 30, 37, 60, 65 and 95°C /26/. The kinetic data of hydroperoxides accumulation in the presence of RHgX, $R_2Hg$ at 37 and 60°C demonstrate the principal difference in the mechanistic mode of organomercurials action in oleic acid oxidation /25/.

The main products of R'H oxidation are highly reactive species $R'OO^•$ and R'OOH /28/. Fig. 1 presents possible pathways of the organometallic compounds reactivity towards these intermediates. The concurrence between the interaction of $R_nMX_m$ with either peroxyl radicals or hydroperoxides is expected to be a cause of their different action. Indeed at 37°C the accumulation of R'OOH is a slow process (rate constant ~

$(0{,}3 \div 0{,}8) \cdot 10^{-4}\ s^{-1}$). The concentrations of R'OOH and RHgX, $R_2Hg$ are comparable, and the organomercury compounds interact with R'OOH in protolytic C-M bond's cleavage reactions /28/ (Eq. 1,2), confirmed by using IR, NMR and UV-vis experiments and by the monitoring of MDA accumulation /24,25/:

$$RHgX + R'OOH \rightarrow RHgOOR' + HX \quad (1)$$

$$R_2Hg + R'OOH \rightarrow RHgOOR' + RH \quad (2)$$

The relative change in R'OOH concentrations ($A_i$) in the presence of organomercurials at temperature 37°C is ~ 50% of the corresponding values for substrate's autooxidation. On the other hand the value of Ai for carbonyl compounds accumulation in the presence of organomercurials shows a 3-4 time increase when compared with the corresponding values of substrate autooxidation. Therefore at 37°C RHgX, $R_2Hg$ have a prooxidative activity. At temperature > 50°C the rate constants for R'OOH formation are higher than the corresponding parameters for R'OOH decomposition. In this region RHgX, $R_2Hg$ interact with the excess of active oxygen-centered peroxyl radicals $R'OO^\bullet$ in $S_H2$ reaction /30/ (Eq. 3,4).

$$RHgX + R'OO^\bullet \rightarrow R^\bullet + R'OOHgX \quad (3)$$

$$R_2Hg + R'OO^\bullet \rightarrow R^\bullet + R'OOHgR \quad (4)$$

The $S_H2$ process might be synchronic or might include the formation of the metal-centered radical intermediate (Eq. 5,6).

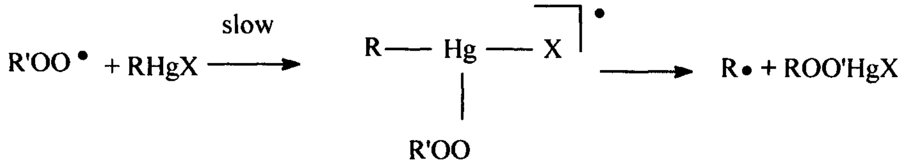

(5)

$$R'OO^\bullet + R_2Hg \rightarrow [(R'OO)R_2Hg]^\bullet \rightarrow (R'OO)HgR + R^\bullet \quad (6)$$

The loss of R group during the oxidative destruction of $(C_6H_5)_2Hg$ in oleic acid has been confirmed by using IR spectroscopy /25/. Fig. 2 represents a change of the absorption bands corresponding to the Hg-C bond frequencies at 400-500 $cm^{-1}$. The initial spectrum shows the absorption band at 462 $cm^{-1}$ (Fig. 2, a) which correspond to C-Hg bond in disubstituted organomercury compound $R_2Hg$. The loss of the intensity of this band and appearance of a new band at 453 $cm^{-1}$ (Fig. 2, b-d) which corresponds to the monosubstituted derivative of mercury proves the formation of RHgX according to Eq.4.

**Table 1.**

The kinetic data for the oxidation of oleic acid in the presence of organomercury and organotin compounds*

| Additives | $37^0C$ | | $60^0C$ | |
|---|---|---|---|---|
| | $k\cdot10^{-4}, s^{-1}$ | $A_i$ | $k\cdot10^{-4}, s^{-1}$ | $A_i$ |
| - | 1.11±0.06 | 1 | 2.42±0.08 | 1 |
| $CH_3HgI$ | 0.94±0.07 | 0.74 | 3.01±0.15 | 1.85 |
| *n*-$C_3H_7HgBr$ | 0.93±0.07 | 0.72 | 2.64±0.09 | 1.34 |
| *i*-$C_3H_7HgBr$ | 0.85±0.06 | 0.59 | 2.27±0.17 | 1.33 |
| $C_6H_5HgBr$ | 0.82±0.05 | 0.56 | 2.61±0.06 | 1.29 |
| $(C_6H_5)_2Hg$ | 0.76±0.04 | 0.48 | 3.44±0.21 | 3.77 |
| $CH_3C_6H_4HgBr$ | 0.8±0.05 | 0.54 | 2.48±0.07 | 1.08 |
| $(CH_3C_6H_4)_2Hg$ | 0.74±0.03 | 0.48 | 3.33±0.19 | 3.14 |
| $CH_3SnCl_3$ | 1.1±0.04 | 1.43 | 2.93±0.05 | 1.22 |
| $(CH_3)_2SnCl_2$ | 1.3±0.03 | 1.61 | 2.95±0.16 | 1.25 |
| $(CH_3)_3SnCl$ | 1.5±0.03 | 3.27 | 3.21±0.11 | 1.3 |
| $C_2H_5SnCl_3$ | 1.04±0.02 | 2.41 | 3.3±0.11 | 1.43 |
| $(C_2H_5)_2SnCl_2$ | 1.05±0.05 | 1.72 | 3.3±0.07 | 1.45 |
| $(C_2H_5)_3SnCl$ | 1.4±0.03 | 2.45 | 3.33±0.09 | 1.45 |
| $(n\text{-}C_4H_9)_2SnCl_2$ | 1.01±0.01 | 2.62 | 3.31±0.11 | 1.4 |
| $(n\text{-}C_4H_9)_3SnCl$ | 1.09±0.05 | 1.45 | 3.4±0.15 | 1.55 |
| $C_6H_5SnCl_3$ | 1.05±0.03 | 1.54 | 3.15±0.13 | 1.03 |
| $(C_6H_5)_2SnCl_2$ | 1.29±0.04 | 2.52 | 3.25±0.13 | 1.38 |
| $(C_6H_5)_3SnCl$ | 1.33±0.04 | 2.55 | 3.42±0.09 | 1.56 |

*k – rate constants of pseudo-first order reaction of R'OOH accumulation; $A_i$ – concentration's change when compared to the control experiment of the substrate autooxidation: $A_i = [(C_i\text{-}C_oi)/C_oi] : [(C_k\text{-}C_ok)/C_ok$, where Ci and $C_i$ – the initial and final (after 5 h) concentrations of R'OOH in the presence of additives respectively; $C_k$ и $C_ok$ - the initial and final (after 5 h) concentrations of R'OOH in control experiment respectively.

The homolytic cleavage of C-Hg bond leads to the generation of reactive organic radicals R• that serve as initiators in the free radical chain reaction. The main pathway for free organic radicals is the participation in the initiation step (H• abstraction from the substrate molecule R'H).

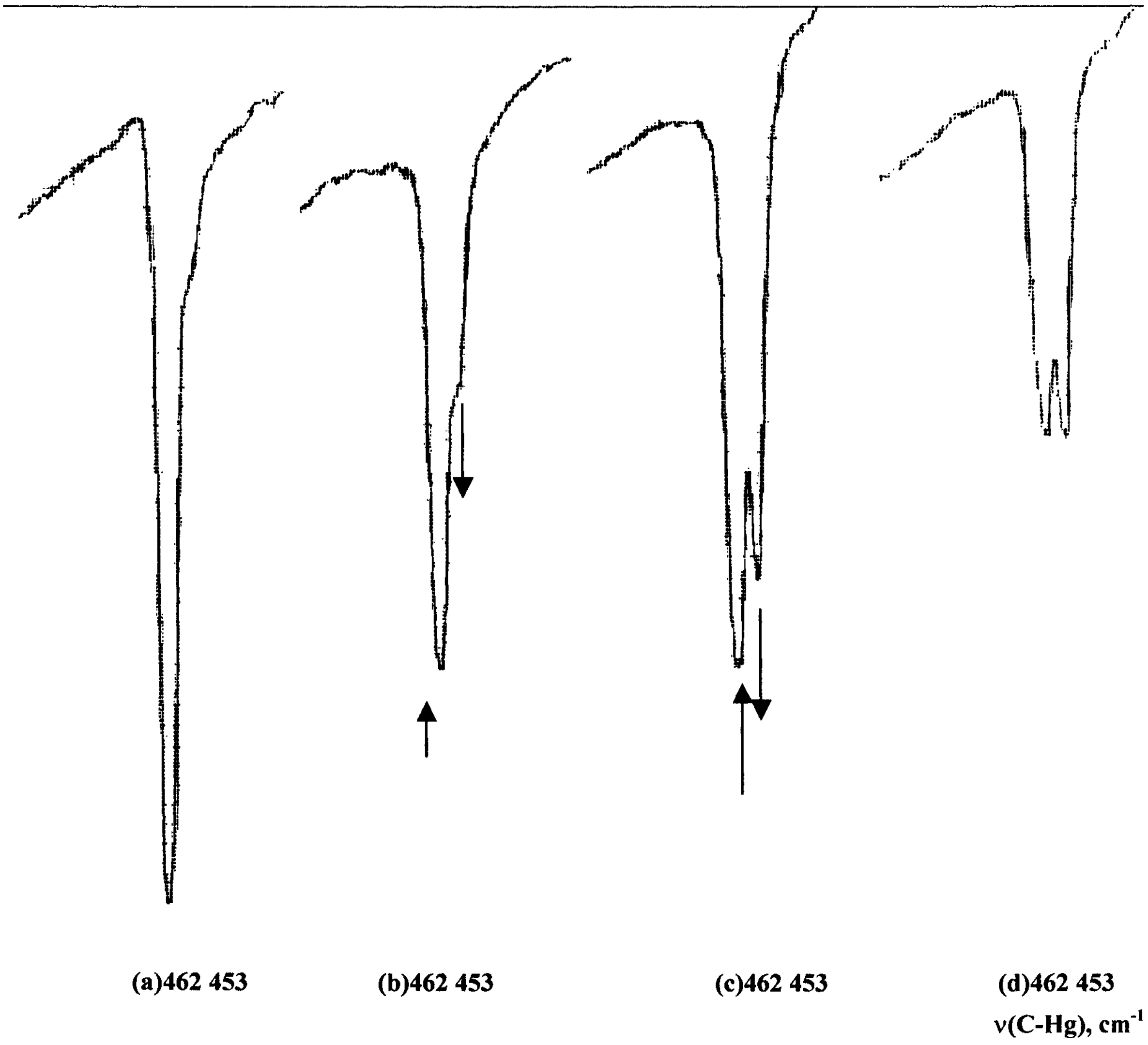

**Fig. 2:** The change of ν(C-Hg) frequencies in $(C_6H_5)_2Hg$. Experimental conditions: oleic acid in the presence of 1.53 mM $(C_2H_5)_2Hg$ and oxygen at 20°C.

The promoting effect of $R_nSnX_{4-n}$ upon the R'OOH formation depends on temperature as well. Despite the fact that the increase of R'OOH formation has been observed in the whole range of temperatures investigated the mechanistic study confirms the assumption of their different mode of reactivity /26/. The bimolecular radical substitution $S_H2$ reaction at tin atom can proceed quite easily /31/. The formation of intermediate five-coordinated Sn species containing radical is preferable when compared with three-coordinated Hg (Eq. 5-8).

$$R'OO\bullet + R_nSnX_{4-n} \rightarrow R_{n-1}SnX_{4-n}(OOR') + R\bullet \quad (7)$$

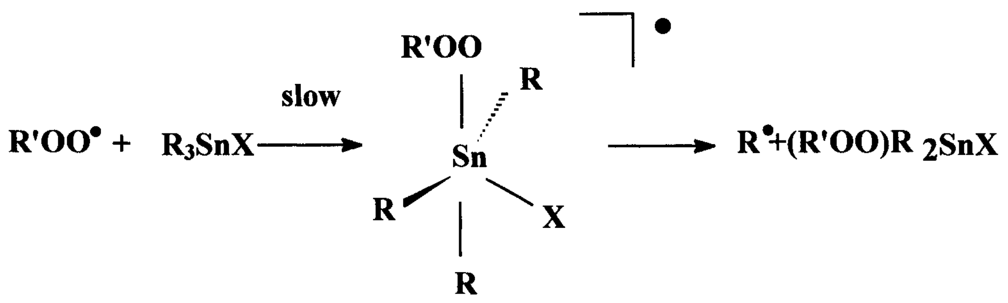

(8)

The R'OOH formation rate constants *k* and the values of R'OOH concentrations relative changes $A_i$ increase with the number of R groups in $R_nSnX_{4-n}$ at 37°C, as can be clearly seen in Table 1. However at 60°C the rates of hydroperoxides formation and the rate of their decomposition become close; kinetic curves form typical for radical chain processes, and the participation of $R_nSnX_{4-n}$ in the interaction with R'OOH seems to play an important role (Eq. 9).

$$R'OOH + R_nSnX_{4-n} \rightarrow R_nSnX_{3-n}(OOR') + HX \quad (9)$$

At 90°C protolytic decomposition of $R_nSnX_{4-n}$ is a major process and the dependence of R'OOH accumulation on the number of R groups in $R_nSnX_{4-n}$ takes the opposite character. Fig. 3 presents the kinetic curves of R'OOH accumulation in the presence of ethyl derivatives of tin.

The reactions (1-9) involve the formation of organometallic peroxides which are unstable and may give various radical intermediates /31/ – promoters of further radical chain processes. Moreover the generation of metal-centered radicals that interact easily with dioxygen to produce oxygen-centered radicals is also expected. The reactions of the membrane-active $(n\text{-}C_4H_9)_3SnX$ (X = $OCH_3$, Cl, Br, I) with $O_2^{\bullet -}$ have been investigated in the aprotic solvent system [cis-dicyclohexano-18-crown-6 ether / DMSO] using ESR /32/. Conductivity measurements of $(n\text{-}C_4H_9)_3SnX$ solutions demonstrate that these compounds dissociate to produce $(n\text{-}C_4H_9)_3Sn^+$ cations which interact with $O_2^{\bullet -}$ to give (μ-superoxo)bis(tributylstannyl) radicals. The authors /32/ proposed that μ-superoxo radical complexes play the key role in the initiation of lipid peroxidation processes *in vivo*.

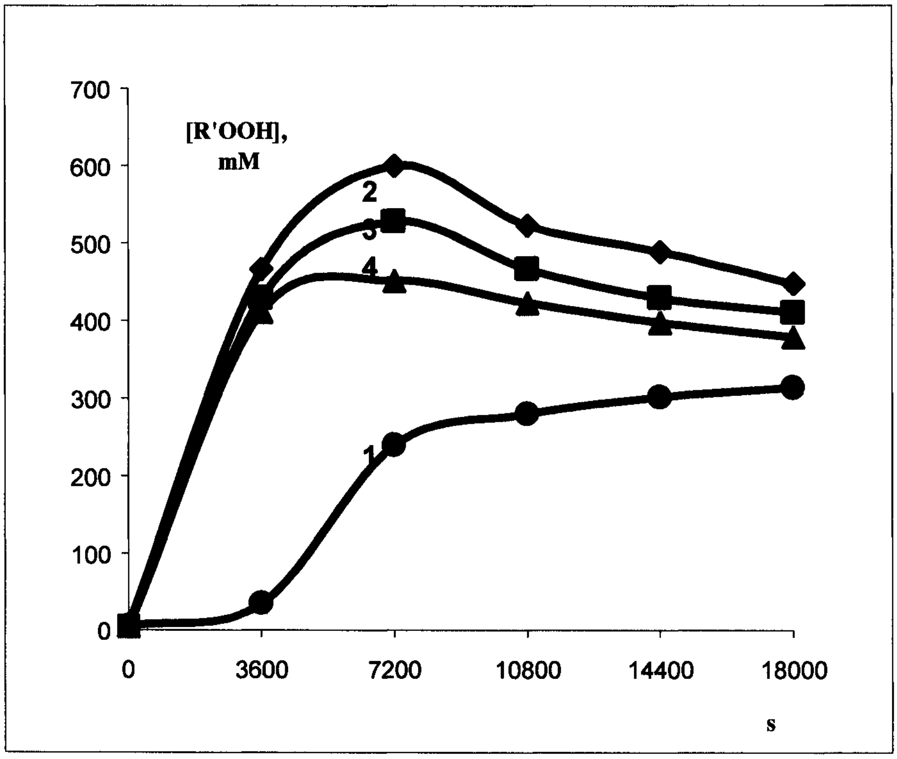

**Fig. 3:** Curves for R'OOH formation at 90°C in the presence of 1 mM $(C_2H_5)_nSnCl_{4-n}$: oleic acid without akdditives (1), in the presence of $(C_2H_5)SnCl_3$ (2), $(C_2H_5)_2SnCl_2$ (3), $(C_2H_5)_3SnCl$ (4).

### THE IMPACT OF ORGANOMETALLICS ON PHOSPHOLIPIDS

The explanation of the biochemical mode of organometallic compounds action (including organic derivatives of Hg and Sn) follows the concept of the interaction of metal center with electron-donor heteroatoms in biologically important substrates /1,5,33/. From this point of view, phosphoryl-containing fragments, e.g. anionic phosphodiester groups ($OPO^-$), are important moieties in phosphor- and phosphono-lipids /34/. From the study of the dimer of bis[(di-*n*-butyl-3,6-dioxaheptanoato)tin] and tri-*n*-butyltin-3,6,9-trioxodecanoate with calf thymus DNA samples it was proposed that both organotin compounds do interact with DNA, probably at the level of the phosphate groups /35/. The ability of phenyltin compounds to induce structural changes in the phosphatidylcholine bilayers has been studied by NMR $H^1$and $P^{31}$ spectroscopy in the presence of dodecyltrimethylammonium chloride, bromide and iodide. The presence of the surfactant influences the interaction of phenyltins with model membranes and the changes are dependent on the kind of the counter-ion /36/. The alteration in lipid profiles induced by mercury has been shown to be time-dependent /17/. The content of phospholipids in brain of catfish enhanced significantly at 30 days. Liver exhibited elevated levels of total lipids, phospholipids and cholesterol. By using human HL-60 leukemia cells, it was shown that $(n\text{-}C_4H_9)_2SnCl_2$ and $(CH_3)_3SnCl$ induce arachidonic acid liberation or its rearrangement within the phospholipids before a loss in viability can be detected and an increase of free fatty acid and eicosanoids before cell death could be detected /37/. Primarily affected is phosphatidylethanolamine which loses arachidonic acid and, to a minor extent, phosphatidylcholine. The study oriented towards the definition of the mechanism by which $CH_3HgCl$ induces human T-cell apoptosis shows that cardiolipin, a mitochondrial phospholipid, was oxidized /38/.

The complexation of $R_nSnX_{4-n}$ (n = 1-3) with phosphodiester fragments of the lipid bilayer might be the key step in the interaction of organotins with cell membranes. In that case phospholipids are supposed to play a protective role, preventing toxic organotin compounds from penetrating the cells and intracellular components. However, this mechanism does not include the possible carbon to metal bond cleavage.

In order to prove the assumption that the formation of reactive radical species following the homolytic cleavage of C-Sn bond might be the cause of a toxic action of $R_nSnX_m$ the synthesis of organotin complexes with phosphocholine (PC) and dimyristoyl-L-α-phosphatidylcholine DMPC) as model structural components of lipids has been achieved and their influence on the oleic acid peroxidation has been studied /27,39/.

Figs. 4a, 4b show the kinetic curves of the R'OOH accumulation in the presence of methyl derivatives of tin $(CH_3)_nSnCl_{4-n}$ and their complexes with phosphocholine $[(CH_3)_nSnCl_{4-n}]_k$•PC at 37°C. The coordination of organotin molecules to the phosphocholine moieties does not influence significantly the accumulation of oleic acid hydroperoxides. This experimental fact allows suggesting that the first step of organotins interaction with lipids fragment does not prevent the activity of these compounds in radical processes of lipids peroxidation. Therefore the effect of organotin compounds on cellular membranes may be dependent upon both the complexation with membrane fragments and the participation in radical/oxidative processes that leads to the disturbance of lipids bilayer and membrane damage.

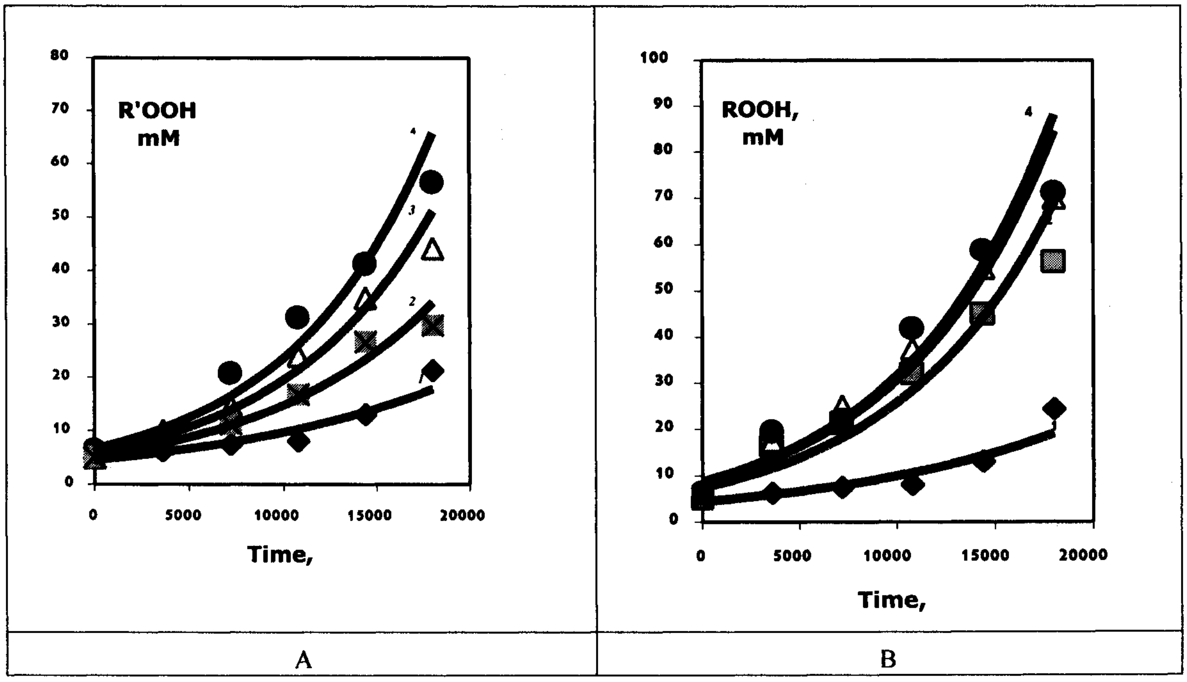

**Fig. 4:** Curves for R'OOH formation at 37°C in the presence of 1 mM $(CH_3)_nSnCl_{4-n}$ (a) and $[(CH_3)_nSnCl_{4-n}]_k$•PC (b): oleic acid without additives (1a), (1b); in the presence of $CH_3SnCl_3$ (2a), $CH_3SnCl_3$•PC (2b); $(CH_3)_2SnCl_2$ (3a), $(CH_3)_2SnCl_2$•PC (3b); $(CH_3)_3SnCl$ (4a), 0,5 mM $[(CH_3)_3SnCl]_2$•PC (4b).

### LIPID PEROXIDATION IN THE PRESENCE OF ORGANOMETALLICS *IN VITRO* AND *IN VIVO*

The data illustrated with the examples of organomercury- and organotin-mediated oxidative damage to the principal components of the membrane bilayers, confirm the mechanistic concepts on the capacity of RHgX, $R_2Hg$, $R_nSnX_{4-n}$ to generate reactive oxygen species and other active organic intermediates under physiological conditions /40/. Their redox and radical reactivity may underlay the mechanism of mediation of oxidative damage to cell constituents. Treatment of the hypothalamic neural cells with methylmercury salt (10 μM for 3 h and 5 μM for 24 h) results in increased formation of ROS, and 20% and 56% cell death respectively /41/. These data suggest that methylmercury-mediated cell killing correlates closely with ROS generation. A significant increase in the ROS formation rate in rat brain was detected 7 days after the injection of $CH_3HgX$ (5 mg/kg) /42/. The formation of oxygen-centered radical species has several biochemical consequences; some of them include the interaction of ROS with carbon- or metal-centered radicals formed in biodegradation of $R_nMX_m$.

An *in vivo* and *in vitro* comparative analysis of the influence of methyl mercury salts on the lipid peroxidation as a biomarker of the oxidative stress of the organism has been performed. The *in vivo* experiments with rats as testing organisms show the promotion of lipids peroxidation monitored by TBARS (MDA) concentration's increase when rats were pretreated by intraperetonial injection with $CH_3HgNO_3$ (5 mg/kg weight) /43/ (Table 2).

**Table 2.**

Lipids peroxidation level in rat liver homogenates in vivo in the presence of methylmercury nitrate*

| Additive | Haemolysis level | Lipids peroxidation level | | |
|---|---|---|---|---|
| | % | **Enzymatic peroxidation, MDA, nM·h$^{-1}$** | **Non-enzymatic peroxidation, MDA, nM·h$^{-1}$** | **Non-enzymatic peroxidation**, MDA, nM** |
| - | 0.33±0.186 | 18.14±3.1 | 57.51±15.4 | 2.08±0.4 |
| $CH_3HgNO_3$ | 1.117±0.29 | 28.66±1.39 | 98.13±17.45 | 4.5±0.4 |

* Experimental conditions: tested animal weight was 331,25±15 g and 264,5±35 g in control experiment and in the presence of methylmercury nitrate correspondingly; 4 animals were investigated in each group of experiments; enzymatic peroxidation level was measured as spontaneous process, non-enzymatic peroxidation level was measured by addition of ascorbic acid and More salt; haemolysis of erythrocytes was monitored by addition of $H_2O_2$. ** Non-enzymatic peroxidation level was measured after addition of $CCl_3COOH$.

The *in vitro* experiments with rat liver homogenates show that $CH_3HgI$ stimulates lipids peroxidation monitored by increase in MDA (Table 3).

**Table 3**

Lipids peroxidation level in rat liver homogenates in vitro in the presence of 1 mM methylmercury iodide*

| **Samples** | **Lipids peroxidation level, MDA, nM** | |
|---|---|---|
| **Additive** | - | $CH_3HgI$ |
| 1 | 0.94±0.13 | 2.46±0.12 |
| 2 | 1.77±0.08 | 4.24±0.1 |
| 3 | 1.94±0.03 | 4.22±0.55 |
| 4 | 2.1±0.04 | 4.25±0.1 |
| 5 | 2.54±0.2 | 4.43±0.1 |
| 6 | 2.8±0.03 | 4.46±0.3 |
| 7 | 3.22±0.14 | 6.3±0.03 |
| 8 | 5.26±0.05 | 6.63±0.32 |
| 9 | 5.56±0.09 | 6.75±0.12 |
| 10 | 6.33±0.85 | 12.50±0.89 |
| 11 | 7.66±0.6 | 13.74±0.14 |
| 12 | 7.81±0.79 | 11.3±0.6 |

The lipid peroxidation level in liver of fish samples of Russian sturgeon (*Acipenser guendelstaedti B.*) was studied in the presence of $(CH_3)_3SnCl$ (Table 4) and at various concentrations of $CH_3HgI$ (Fig. 5) /44/ as a biomarker of adaptation potential of the organism /45/.

**Table 4**

The peroxidation level in fish liver homogenates of *Acipenser guendelstaedti B.* when treated orally with $CH_3HgI$ and $(CH_3)_3SnCl$*

| Samples** | MDA, nmol | |
|---|---|---|
| | Without additives | $(CH_3)_3SnCl$ |
| 1 | 1.32 ± 0.08 | 4.52 ± 0.17 |
| 2 | 1.33 ± 0.07 | 7.50 ± 0.12 |
| 3 | 1.34 ± 0.06 | 2.53 ± 0.09 |
| 4 | 1.33 ± 0.07 | 3.23 ± 0.09 |
| 5 | 1.33 ± 0.06 | 3.24 ± 0.07 |

*Additives content 150 mg·kg$^{-1}$ fodder; **6 fish samples, 5 experiments per one sample

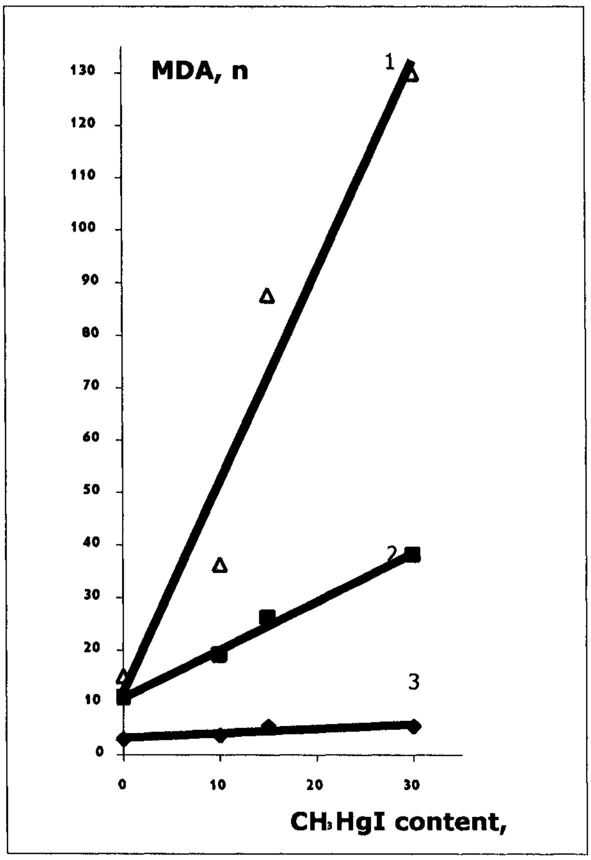

**Fig. 5:** Dependence of lipids peroxidation level *in vivo* in liver homogenates of Russian sturgeon (*Acipenser guendelstaedti B.*) on the content of $CH_3HgI$: (1) non-enzymatic peroxidation level was measured by addition of ascorbic acid and More salt; (2) enzymatic peroxidation level was measured as spontaneous process, (3) non-enzymatic peroxidation level was measured after addition of $CCl_3COOH$.

### NATURAL AND SYNTHETIC ANTIOXIDANTS AS ANTIDOTES AGAINST ORGANOMETALLICS TOXIC EFFECTS

The involvement of lipophylic organometallic compounds in cellular radical and redox processes means the promotion of membrane bilayer oxidative destruction due to the generation of ROS and other active radical species. These events might be prevented or inhibited by the antioxidants present in living organisms. Cellular self-defense response is manifested by increase of the antioxidant enzyme activities (glutathione peroxidase, glutathione reductase, glutathione transferase, superoxide dismutase, catalase, etc.). However there is strong evidence that intoxication with organic derivatives of Hg and Sn causes a disturbance in the antioxidative defense system as well /6,11/.

It is a well-known fact that methylmercury cation $CH_3Hg^+$ interacts with glutathione in human blood, leading to glutathione level depletion and enhancement of oxidative stress /46/. Moreover, the decrease of vitamins E and C contents was observed, for instance, in rat kidney after 12 h administration of mercury compounds /47/. The changes in glutathione-dependent enzyme activities were studied as effects of oral exposure to $(C_6H_5)_3SnOCOCH_3$ for 70 days on hepatic and renal enzymes involved in glutathione metabolism in rabbits and lambs /48/. The inhibition of glutathione S-transferases isolated from larval midguts, *Spodoptera frugiperda*, by $(C_6H_5)_3SnCl$ has been detected /49/. The authors suggest that the depression of glutathione S-transferase and glutathione peroxidase is one part of the complex mechanism of organotins cytotoxicity. By using a flow cytometry study it was shown that n-$C_4H_9)_3SnCl$ induces a change in cellular level of glutathione in rat thymocytes /50/.

Since the mechanism of glutathione level decrease by $R_nMX_m$ is supposed to include the interaction of metal with S atoms, the sulfur-containing agents can be used as antidotes. Among them dimercaptocompounds (2,3-dimercaptopropane-1-sulfonic acid, meso-2,3-dimercaptosuccinic acid, diethyldithiocarbamate, monoisoamyl meso-2,3-dimercaptosuccinate) show a significant protective effect on the toxicity of organotins and organomercurials /51-53/.

On the other hand the cascade of radical reactions following the accumulation of active species formed due or from the organometallics biodegradation activates chain radical lipids peroxidation. Therefore the natural or synthetic inhibitors may serve as detoxificating agents. The first experimental data showing the protective effect of vitamin E was presented early /54/, accompanying the proposition that free radicals derived from methylmercury compounds are responsible for oxidative stress.

Indeed, when enhanced lipid peroxidation in liver, kidney and brain of mice has been observed after exposure to $CH_3HgCl$, the pre-treatment with diets containing α-tocopherol or β-carotene produces protective effect /15/. High dietary α-tocopherol protected against $CH_3HgCl$ induced hepatic lipid peroxidation and increased the activity of total glutathione peroxidase and Se-dependent glutathione peroxidase inhibited by $CH_3HgCl$ in the kidneys. Natural antioxidants – vitamin E and ascorbic acid – may protect against *in vivo* toxic effects of mercury in the mammalian tissues /55,56/. The *in vivo* effectiveness of 6-hydroxy-2,5,7,8-tetramethylchroman-2-carboxylic acid, used as water soluble vitamin E analog – antioxidant "Trolox", against the methylmercury-induced cellular responses was demonstrated /57/.

The addition of vitamin E (α-tocopherol) to the diet containing $CH_3HgNO_3$ demonstrates the inhibition of peroxidation level in rat liver homogenates /43/ (Table 4).

**Table 4**

The impact of methylmercury iodide and vitamin E on the lipid peroxidation level in rat liver homogenates*

| **Samples** | *Lipids peroxidation level, MDA, nM* | | | |
|---|---|---|---|---|
| | **Without α-tocopherol** | | **With α-tocopherol** | |
| | - | $CH_3HgNO_3$ | - | $CH_3HgNO_3$ |
| 1 | 1.24±0.15 | 2.52±0.1 | 0.38±0.07 | 0.56±0.06 |
| 2 | 4.53±0.14 | 6.44±0.22 | 2.26±0.14 | 2.91±0.14 |
| 3 | 2.5±0.15 | 4.54±0.03 | 1.13±0.04 | 1.67±0.09 |
| 4 | 3.86±0.09 | 6.09±0.05 | 2.53±0.06 | 2.89±0.02 |

* Rats were pretreated by intraperitoneal injection with $CH_3HgNO_3$ (5 mg/kg weight) or with $CH_3HgNO_3$ (5 $mg{\cdot}kg^{-1}$ weight) and α-tocopherol (15 $mg{\cdot}kg^{-1}$ weight)

The monitoring has been done by studying the enzymatic and non-enzymatic peroxidation levels. The detoxification effects of α-tocopherol on the enzymatic and non-enzymatic peroxidation level *in vivo* in fish samples (Stellate sturgeon, Russian sturgeon) were observed when tested fish were fed with methyl derivatives of Hg and Sn /58/. (Figs. 6,7).

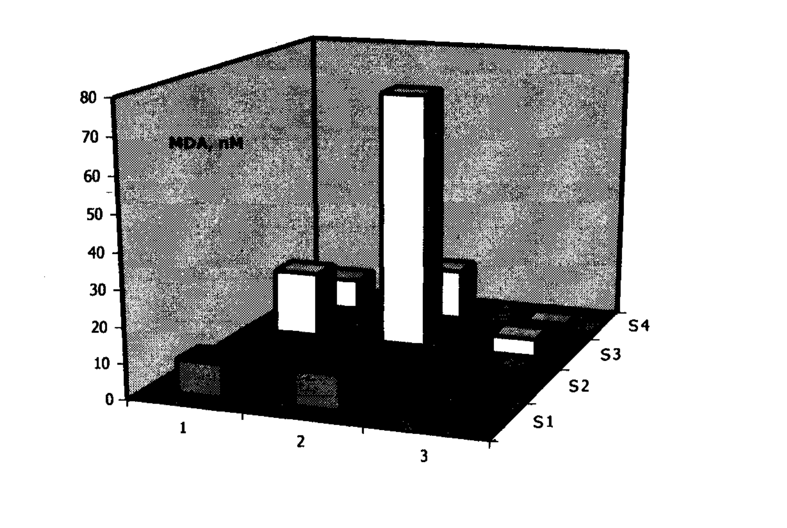

**Fig. 6:** Impact of $CH_3HgI$ (4 $mg{\cdot}kg^{-1}$ weight) and α-tocopherol (10 $mg{\cdot}kg^{-1}$ weight) on lipids peroxidation level in vivo in liver homogenates of Stellate sturgeon: (1) non-enzymatic peroxidation level was measured by addition of ascorbic acid and More salt; (2) enzymatic peroxidation level was measured as spontaneous process, (3) non-enzymatic peroxidation level was measured after addition of $CCl_3COOH$; (P1) control, (P2) addition of α-tocopherol (10 $mg{\cdot}kg^{-1}$ weight), (P3) addition of $CH_3HgI$ (4 $mg{\cdot}kg^{-1}$ weight), (P4) addition of $CH_3HgI$ (4 $mg{\cdot}kg^{-1}$ weight) and α-tocopherol (10 $mg{\cdot}kg^{-1}$ weight).

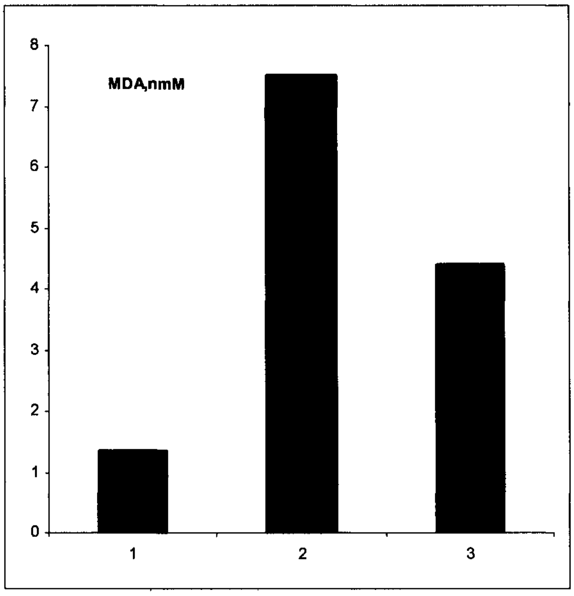

**Fig. 7:** Impact of $(CH_3)_3SnCl$ and α-tocopherol (150 mg·kg$^{-1}$ fodder) on lipids peroxidation level *in vivo* in liver homogenates of Russian sturgeon: (1) control, (2) addition of $(CH_3)_3SnCl$ (150 mg·kg$^{-1}$ fodder), (P4) addition of $(CH_3)_3SnCl$ (150 mg·kg$^{-1}$ fodder) and α-tocopherol (150 mg·kg$^{-1}$ fodder).

The experiments with model compound – oleic acid, as a representative of unsaturated fatty acids, proved the assumption of the preventive effect of antioxidants on the prooxidative function of organomercury (Table 5,6) and organotin compounds (Fig. 8) /24,26/.

**Table 5**

The kinetic data for the oxidation of oleic acid in the presence of 1 mM organomercury compounds and 1 mM antioxidants at 60°C*

| Additives | k·10$^{-4}$, s$^{-1}$ | | |
|---|---|---|---|
| | **Without additives** | **$CH_3HgI$** | **$C_6H_5HgBr$** |
| **Without additives** | 2.42±0.08 | 3.01±0.15 | 2.61±0.06 |
| **α-tocopherol** | 1.28±0.05 | 1.89±0.02 | 1.67±0.12 |
| **α-tocopherol acetate** | 1.66±0.12 | 2.16±0.01 | 1.88±0.14 |
| **2,4,6-tri-*tert*-butylphenol** | 1.03±0.12 | 1.12±0.02 | 1.29±0.09 |
| **2,6-di- *tert*-butylphenol** | 1.73±0.15 | 2.47±0.01 | 2.08±0.14 |

*k – rate constants of pseudo-first order reaction of R'OOH accumulation

The data presented in Fig. 8 demonstrates the strong dependence of the R'OOH accumulation rate on the number of ethyl groups in the organotin molecule. The values of $k_i/k_o$ (where $k_i$ – R'OOH formation rate constants in the presence of $(C_2H_5)_nSnCl_{4-n}$ and antioxidant, $k_o$ - R'OOH formation rate constants without additives) are 0.68, 0.56 and 0.52 for $(C_2H_5)_3SnCl$, $(C_2H_5)_2SnCl_2$ and $(C_2H_5)SnCl_3$ respectively /26/.

Since the key reaction of the active radicals with antioxidant molecule is the abstraction of H atom from the phenol, the use of equimolar mixture of $R_nSnCl_{4-n}$ and 2,6-di-*tert*-butylphenol proves the participation of organic radicals derived from the C-Sn bond cleavage.

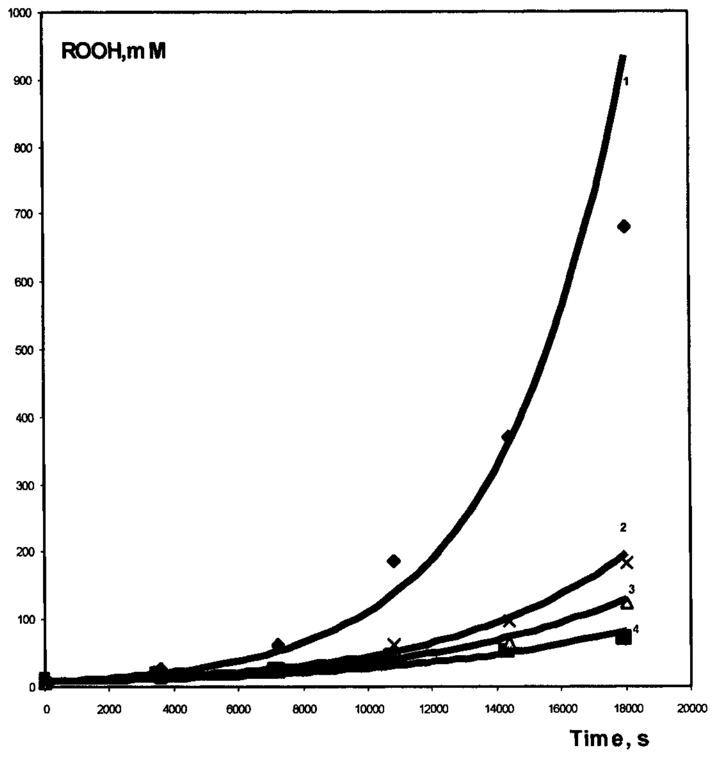

**Fig. 8:** Curves for R'OOH formation at 65°C in the presence of 1 mM $(C_2H_5)_nSnCl_{4-n}$ and 1 mM 2,6-di-*tert*-butylphenol: oleic acid without additives (1), in the presence of $(C_2H_5)_3SnCl$ (2), $(C_2H_5)_2SnCl_2$ (3), $(C_2H_5)SnCl_3$ (4).

This consideration was supported by the comparison of the rate constants of the $S_H2$ reactions (Equation 3,7) and H abstraction from phenolic antioxidats when using model compounds 3,5-di-*tert*-butyl-4-hydroxyphenylmercury chloride (A) and bis-(3,5-di-*tert*-butyl-4-hydroxyphenyl)tin dichloride (B) containing both antioxidant fragment and metal atom /59/.

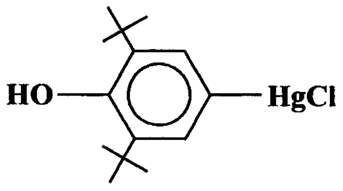

A

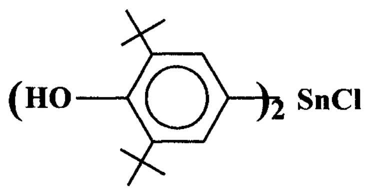

B

Figs. 9,10 present the antioxidative activity of phenolic derivatives of Hg and Sn (A,B) in the oxidation of both oleic acid and methyl oleate.

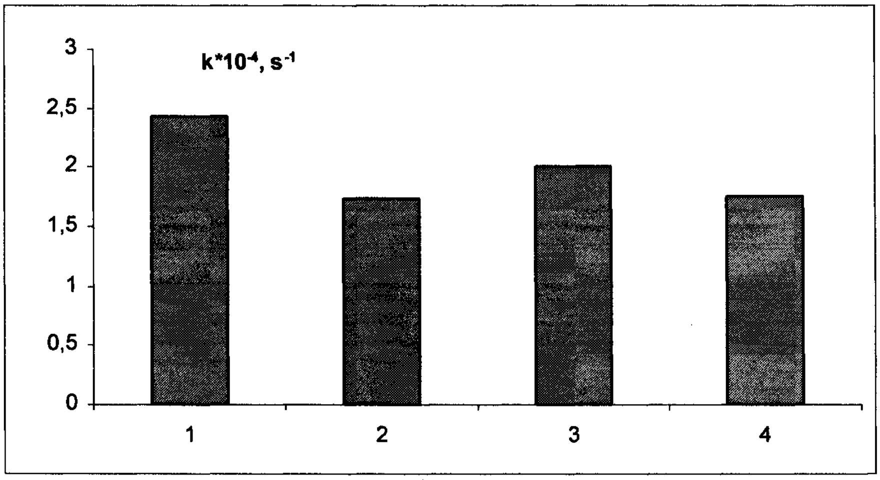

**Fig. 9:** R'OOH formation rate constants in the oxidation of oleic acid at 60°C: oleic acid without additives (1), in presence of 1 mM 2,6-di-*tert*-butylphenol (2), in presence of 1 mM 3,5-di-*tert*-butyl-4-hydroxyphenylmercury chloride (A) (3), in presence of 1 mM 2,6-di-*tert*-butylphenol and 1 mM 3,5-di-*tert*-butyl-4-hydroxyphenylmercury chloride (A) (4).

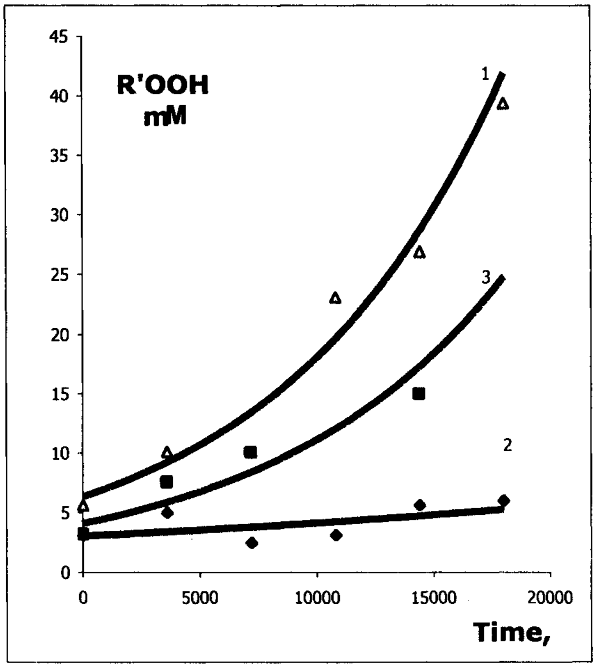

**Fig. 10:** Curves for R'OOH formation in the oxidation of methyl oleate at 50°C: methyl oleate without additives (1), ), in presence of 1 mM 2,6-di-*tert*-butylphenol (2), in the presence of 1 mM bis-(3,5-di-*tert*-butyl-4-hydroxyphenyl)tin dichloride (B) (3).

These data make it possible to propose that the rates of radical substitution reactions at metal center are lower than the corresponding values of hydrogen abstraction by peroxyl radicals of substrates. Nevertheless the homolytic cleavage of carbon to metal bonds might play a significant role in the mechanism of organometallics action in the radical and oxidative bioprocesses.

### CONCLUSION

The toxicity mechanisms of mercury and tin organic derivatives are still a matter for debate. The explanations of their biochemical mode of action are inconsistent. The organometallic compounds $R_nMX_m$ (M = Hg, Sn) are broad-spectrum biocidal agents whose toxic effect is primarily manifested at the membrane level due to the lipophilic nature of their molecules. The ability of mercury and tin atoms to bind biologically important molecules through the interaction with heteroatoms of biosubstrates and to disturb mostly protein systems is well known. On the other hand the involvement of $R_nMX_m$ in the biochemical reactions in which the organic moieties and carbon to metal bonds are responsible for the key processes is still purely investigated. However the dependence of the organometallics toxicity on the type and number of R groups in their molecules is clearly proved.

The data collected recently and presented here are fundamentally important to recognizing the difference between the role of metal centers and of organic fragments in the biochemical behavior of $R_nMX_m$ in their interaction with primary biological targets when entering a living organism and penetrating a cellular membrane. Toxic doses of organomercury and organotin compounds are capable of disturbing the natural oxidation/reduction balance in cells through various mechanisms stemming from their own complex radical and redox reactions with endogenous oxidants. The consequences of this action produce the effects on cellular antioxidant systems. The resulting oxidative stress may damage cellular membranes and membrane-dependent redox sensitive enzymatic systems. This, in turn, may produce a variety of toxic effects, including pathological processes that lead to cell death.

Therefore there is a strong need to investigate in more depth the principal radical bioprocesses which involve the organometallic molecules. The understanding of the mechanistic mode of toxic action of $R_nMX_m$ may lead to new approaches for the utilization not only of chelating agents as antidotes against heavy metals compounds but of inhibitors and antioxidants as preventative additives for the detoxification of heavy metal organic derivatives.

**Acknowledgements:** Financial support by EC (INTAS Program, grant N97-31633), Russian Foundation for Basic Research (grant N03-03-32938), Ministero dell' Istruzione, dell'Universitá e della Ricerca (M.I.U.R., Rome), Universitá di Palermo is gratefully acknowledged.

### REFERENCES

1. Crompton TR (1998) Occurrence and analysis of organometallic compounds in the environment. John Wiley, New York
2. Boening DW (2000) Chemosphere 40: 1335-1351
3. Gadd GM (2000) Science Total Environ 258: 119-127
4. Apostoli P (2002) J Chromat 778: 63-97
5. Stohs SJ, Bagchi D (1995) Free Rad Biol Med 18: 321-336
6. Sarafian TA (1999) In: Sigel A, Sigel H (eds) Metal ions in biological systems. Marcel Dekker, New York 36: 415-444.
7. Gennari A, Viviani B, Galli CL, Marinovich M, Pieters R, Corsini E (2000) Toxicol Appl Pharmacol 169: 185-190
8. Regolia F, Gorbia S, Frenzillib G, Nigrob M, Corsic I, Focardic S, Winstond GW (2002) Mar Environ Res 54: 419–423
9. Hamilton RJ, Kalu C, Prisk E, Padley FB, Pierce H (1997) Food Chem 60: 193-199
10. Mittler R (2002) Trends Plant Sci 7: 405-410
11. Sergent O, Morel I, Cillard J (1999) In: Sigel A, Sigel H (eds) Metal ions in biological systems. Marcel Dekker, New York 36: 251-287
12. Goyer RA (1991) In: Amdur MO, Doull J, Klaassen CD (eds) Casarett and Doull's toxicology: The basic science of poisons. Pergamon Press, New York 629-681
13. Shah DSM, Rojaramani V, Sathick O, Priya RS (2001) J Environ Poll 8: 7-11
14. Mooney HM, Cooney JJ, Baisden CM, Patching JW (1995) Bot Mar 38: 423-429
15. Huang YL, Cheng SL, Lin TH (1996) Biol Trace Element Res 52: 193-206
16. Ferrat L, Romeo M, Gnassia-Barelli M, Pergent-Martini C (2002) Dis Aquat Org 50: 157-160
17. Bano Y, Hasan M (1989) J Environ Sci Health, Part B B24: 145-166
18. Masia A, Avery SV, Sorodu MA, Gadd GM (1998) FEMS Microbiol Lett 167: 321-326
19. Sarapuk J, Kleszczynska H, Przestalski S (2000) Appl Organomet Chem 14: 40-47
20. Meador JP (1993) J Exp Mar Biol Ecol 174: 227-242
21. Radecka H, Zielinska D, Radecki J (1999) Sci Total Environ 234: 147-153
22. Zielinska D, Radecka H, Radecki J (2000) Chemosphere 40: 327-330
23. Chicano JJ, Ortiz A, Teruel JA, Aranda F (2002) J Biochim Biophys Acta 558: 70-81
24. Milaeva ER, Pimenov YT, Berberova NT, Kirillova LB, Gracheva YA, Tyurin VY, Kalyavin VA, Petrosyan VS (2001) Dokl Chem 379: 631-635 (Engl Transl)
25. Milaeva ER, Kirillova LB, Berberova NT, Pimenov YT, Tyurin VY, Grigor'ev EV, Petrosyan VS (2002) Russ J Gen Chem 72: 761-765 (Engl Transl)
26. Petrosyan VS, Milaeva ER, Gracheva YA, Grigor'ev EV, Tyurin VY, Pimenov YT, Berberova NT (2002) Appl Organomet Chem 16: 655-659
27. Petrosyan VS, Milaeva ER, Gracheva YA, Tyurin VY, Grigor'ev EV, Pellerito L (2003) Russ J Org Chem 39: 00-00 (Engl Transl)
28. Porter NA, Mills KA, Carter RL (1994) J Am Chem Soc 116: 6690-6696

29. Niki E (1992) Ando W (ed) Organic Peroxides. John Wiley, New York
30. Russel GA (1989) Acc Chem Res 22: 1-8
31. Davies AG (1997) Organotin Chemistry. Wiley-VCH, New York
32. Rivera JA, Cummings SC, Macys DA (1992) Chem Res Toxicol 5: 698-705
33. Patai S (ed) (1995) The Chemistry of Organic Germanium, Tin and Lead Compounds. Wiley, Chichester
34. Chicano JJ, Ortiz A, Teruel JA, Aranda F (2001) Cell Biol Mol Lett 6: 340
35. Casini A, Messori L, Orioli P, Gielen M, Kemmer M, Willem R (2001) J Inorg Biochem 85: 297–300
36. Rozycka-Roszak B, Pruchnik H (2001) Z Naturforsh C-A J Bioscie 56: 623-628
37. Kafer A, Zoltzer H, Krug HF (1992) Toxicol Appl Pharmacol 116: 125-32
38. Shenker BJ, Guo TL, Shapiro IM (1999) Toxicol Appl Pharmacol 157: 23-35
39. Grigor'ev EV, Pellerito L, Yashina NS, Pellerito C, Petrosyan VS (2000) Appl Organomet Chem 14: 443-448
40. Andersen HR, Andersen O (1993) Pharmacol Toxicol 73: 192-201
41. Suda I, Totoki S, Takahashi H (1991) Arch Toxicol 65: 129-134
42. Huang YL, Cheng SL, Lin TH (1996) Trace Elem Res 52: 193-206
43. Kirillova LB, Berberova NT, Kondratenko EI, Pimenov YT, Milaeva ER, Tyurin VY, Petrosyan VS (2001) Toxicol Vestn N4: 24-28 (Russ)
44. Movchan NO, Pimenov YT, Ponomarev SV, Milaeva ER (2001) Toxicol. Vestn N1: 28-32 (Russ)
45. Braunbeck T, Hinton DE (eds) (1998) Fish ecotoxicology. EXS Series N68. Birkhauser Verlag, Basel
46. Rabenstein DL, Evans CA (1978) Bioinorg Chem 8: 107-114
47. LeBel CP, Ali SF, Bondy SC (1992) Toxicol Appl Pharmacol 112: 161-165
48. Di Simplicio Dchem P, Dacasto M, Carletti M, Giannerini F, Nebbia C (2000) Vet Hum Toxicol 42: 159-162
49. Yu SJ, Abo-Elghar GE Pesticide Biochem Physiol (2000) 68: 173-183
50. Okada Y, Oyama Y, Chikahisa L, Satoh M, Kanemaru K, Sakai H, Noda K (2000) Toxicol Lett Shannon 117: 123-128
51. Merkord J, Weber H, Kroning G, Hennighausen G (2000) Human Experim Toxicol 19: 132-137
52. Nelson-Zlupko L, Dore M.M, Kauffman E, Kaltenbach K, Reyes-Vivas H, Lopez-Moreno F, Chavez E (1996) Compar Biochem Physiol Part C 113: 349-354
53. Belles M, Sanches DJ, Gomez M, Domingo JL, Jones MM, Singh PK (1996) Toxicol 106: 93-97
54. Welsh SO, Soares JH (1976) Nutr Rep Int 13: 43-51
55. Romeo M, Gnassia-Barelli M (1997) Compar Biochem Physiol Part C 118: 33-37
56. Rana SVS, Rekha S, Seema V (1996) Ind J Exp Biol 34: 177–179
57. Patil GR, Rao MV (1999) Ind J Environ Toxicol 9: 53–55
58. Movchan NO, Pimenov YT, Ponomarev SV, Kirillova LB, Milaeva ER (2001) Science for Production 44: 28-29 (Russ)

59. Tyurin VY, Kirillova LB, Gracheva YA, Berberova NT, Pimenov YT, Milaeva ER, Petrosyan VS (2001) Lipids peroxidation in the presence of Hg and Sn organometallic compounds. In: Abstracts, 2001 International Conference on Reaction Mechanisms and Organic Intermediates. P.217

